# Ultrasound-Guided Preoperative Fascia Iliaca Compartment Block for Pain Relief During Positioning for Spinal Anesthesia in Patients With Hip Fracture

**DOI:** 10.7759/cureus.51185

**Published:** 2023-12-27

**Authors:** Neha Nidgundi, Manjula S Rao, Meghna Mukund

**Affiliations:** 1 Anesthesiology, District Hospital Dharwad, Dharwad, IND; 2 Anesthesiology, Yenepoya Medical College, Mangalore, IND

**Keywords:** numeric rating scale (nrs), fascia iliaca compartment block (ficb), spinal anaesthesia, positioning, hip surgery

## Abstract

Background

Fractures around the hip are common in the elderly. For surgical management, the subarachnoid block is the preferred anesthesia technique. Positioning these patients for anesthesia is challenging because of pain. Analgesia in the form of preoperative perineural anesthesia is gaining popularity. We observed the analgesic efficacy of preoperative ultrasound-guided fascia iliaca block, its efficacy during positioning for spinal anesthesia, pain scores, and anesthesiologist comfort while administering spinal anesthesia.

Methodology

An observational study was conducted on patients of 40 to 80 years under the American Society of Anesthesiologists (ASA) physical status I-III, requiring hip surgeries under spinal anesthesia. After pre-anesthetic evaluation, the purpose and protocol of the study were explained to patients, and informed consent was obtained. Pain score using the numeric rating scale (NRS) was recorded. Ultrasound-guided suprainguinal fascia iliaca block was performed using 30 ml of 0.25% levobupivacaine one hour before shifting to the operating room. Pain scores were reassessed. Spinal anesthesia was administered in the operating theatre in a sitting position. Pain during positioning was assessed.

Results

The mean NRS score reduced significantly after ultrasound-guided suprainguinal fascia iliaca block. The mean NRS score was 3.25 during positioning for spinal anesthesia compared to a pre-block score of 9.03, noting a statistically significant reduction (p=0.001).

Conclusion

Fascia Iliaca compartment block (FICB) helps alleviate the pain of hip fractures and makes positioning the subarachnoid block easier.

## Introduction

Hip fractures are among the most common fractures witnessed in the elderly population, which constitutes a great number of orthopedic cases yearly [1]. While the management of these osteoporotic bones is a challenge to the orthopedic surgeon, the anesthesiologist is provided the challenge of managing a geriatric case with multiple comorbid conditions. Of further concern to the anesthesiologist is the perioperative handling of such a patient to position for regional anesthesia or central neuraxial blockade in the setting of severe pain and difficult mobilization. More often than not, nonsteroidal anti-inflammatory drugs (NSAIDs) or opioids provide inadequate analgesia prior to spinal or regional anesthesia and are not ideal for reducing discomfort. This can lead to a prolongation of time taken to deliver anesthesia [2].

Fascia Iliaca compartment block (FICB) is a technique with the intent to block the femoral nerve, lateral femoral cutaneous nerve, and obturator nerve. Evidence suggests that the fascia iliaca compartment block is easy to perform and can be accessed via a minimal-risk approach using ultrasonography. The femoral nerve and fascia iliaca are visualized, and local anesthetic is deposited beneath the fascia, lateral to the femoral nerve. Ultrasound guidance helps visualize anatomical structures better, improves the success rate, quality of the block and onset time, lesser amount of used local anesthetic, and decreases the complications related to landmark technique [3]. Literature analysis shows that FICB provides good pain relief while performing spinal anesthesia with better patient acceptance when performed preoperatively using 0.5% ropivacaine [1,2,4,5]. The drug used in our study was levobupivacaine, which is a long-acting amide local anesthetic agent. Being an S(-) enantiomer of the conventional racemic bupivacaine, it has less cardiovascular and central nervous system toxicity. It acts by reversibly blocking the neuronal intracellular portion of sodium channels and thereby disrupting nerve conduction. Myelinated and small-diameter nerve fibers are blocked rapidly compared to others [6].

This study evaluated the analgesic efficacy of preoperative ultrasound-guided fascia iliaca compartment block to facilitate positioning for spinal anesthesia.

## Materials and methods

Ethical clearance for this study was obtained from Yenepoya Ethics Committee 2 of Yenepoya University, with the reference number YEC2/351 on 31/01/2020. The study was conducted in accordance with the ethical principles outlined in the Declaration of Helsinki. Informed consent was obtained from all the participants, and their confidentiality was strictly maintained throughout the study. 

This was an observational study conducted on 60 patients belonging to the American Society of Anesthesiologists (ASA) physical status I-III, scheduled for surgery for hip fractures under spinal anesthesia. Patients aged 40-80 years with hip fractures (inter-trochanteric fractures, head of femur fractures, sub-trochanteric fractures, or neck of femur fractures) were included in the study. Patients with hemorrhagic diathesis, peripheral neuropathy, allergy to amide local anesthetics, mental disorders, inability to comprehend the NRS pain scale, and uncooperative patients were excluded from the study. All the patients underwent a general physical and systemic examination in the ward one day prior to surgery. The purpose of the study and the methodology were explained to the patient, and written informed consent was taken for fascia iliaca block and spinal anesthesia. Premedication in the form of a tablet of pantoprazole 40 mg and a tablet of alprazolam 0.5 mg was given on the night before surgery.

The patient was shifted to the preoperative room one hour before the procedure. Standard monitors, including pulse oximetry, non-invasive blood pressure, and ECG were attached. An eighteen-gauge IV catheter was placed at the forearm, and 10 ml/kg of ringer lactate solution was infused over 20 minutes. The patient's numeric rating scale (NRS) score was noted. Following skin preparation with a povidone-iodine solution, a sterile high-frequency ultrasound (USG) probe (8-12 MHz) was positioned at the anterior superior iliac spine (ASIS), directed midway between the umbilicus and xiphisternum. The probe was then moved medially along the inguinal ligament to achieve an hourglass pattern.

Subsequently, a short beveled Stimuplex® needle was inserted through the sartorius muscle using an in-plane approach after creating a skin wheal with 1 ml of 0.25% levobupivacaine. The needle entry point was set at 3-4 cm from the transducer's edge, facilitating a reduced needle angle trajectory to the fascial plane and optimizing the ultrasound beam's angle relative to the needle. The needle was advanced until a distinctive pop was felt upon piercing the fascia iliaca. Confirming correct needle placement involved injecting 4-5 ml of 0.9% normal saline, resulting in the appearance of an anechoic fluid collection separating the fascia iliaca from the iliacus muscle. This fluid visibly expanded the compartment at an average depth of 4-6 cm from the skin level. The Stimuplex® needle was further introduced 4 cm into the compartment.

Verification of the needle tip's correct location was accomplished through direct visualization via USG or the accumulation of local anesthetic in the fascia iliaca compartment. Thirty milliliters of 0.25% levobupivacaine was injected, resulting in the separation of fascia from iliacus muscle due to expansion of the fascial compartment due to hydro dissection. NRS score was again assessed at 20 minutes. Sensations over the lateral, medial, and anterior parts of the thigh were checked with the help of cold saline. After one hour, the patient was shifted to the operating room. A subarachnoid block with 3 ml of 0.5% bupivacaine was given in a sitting position. NRS score during positioning for the spinal anesthesia was noted. The anesthesiologist's comfort regarding the patient's positioning was noted. After completing the spinal injection, patients were placed supine.

The data collected was entered into an Excel sheet and analyzed. A paired t-test was applied to test the statistical difference between NRS of pre-block and during positioning.

## Results

In our study, the majority of the patients presenting with hip and proximal femur fractures were between the age group of 60-80 years, and the incidence was seen to be higher in females, i.e., out of 60 patients, 36 were females, and 24 were males as shown in Table 1.

**Table 1:** Demographic details

Demographics	Parameters observed	N	%
Age group	=40	5	8.3
	41 – 50	13	21.7
	51 – 60	10	16.7
	61 – 70	16	26.7
	71 – 80	16	26.7
	Total	60	100.0
Sex	Female	36	60.0
	Male	24	40.0

Pain scores were measured using a numerical rating scale of 0-10, with 0 indicating no pain and 10 representing the worst imaginable pain at various time intervals, as shown in Table 2. It was observed that out of 60 patients, all of them had a pain score of more than seven before the block. Twenty minutes after the block, 10 patients had NRS of more than seven. During positioning, 11 patients had NRS of more than seven. These patients required supplementary analgesia in the form of IV fentanyl 50 mcg at the positioning. On comparing the NRS scores before giving the block and during positioning, the p-value was 0.001, which is highly significant, showing that ultrasound-guided FICB given one hour before the procedure significantly reduces the pain during positioning for spinal anesthesia. 

**Table 2:** The numerical rating scale (NRS) The numerical rating scale (NRS) is a widely used scale to assess pain intensity, where participants rate their pain on a scale typically ranging from 0 to 10, with 0 indicating no pain and 10 representing the worst imaginable pain. The scale provides a quantitative measure of pain experienced by participants. Mean represents the average NRS score within each group. Standard deviation indicates the degree of variability or dispersion of NRS scores around the mean. Number of participants denotes the total count of participants in each group.

NRS	Mean	SD	N	p-value
Pre-block	9.03	0.823	60	
20 min after block	2.77	2.696	60	0.0004
During positioning	3.25	3.266	60	0.001

Sensory blockade in the lateral part of the thigh five minutes after the block was noted in 16.7% of the patients. At 10, 20, and 60 minutes, 38.3%, 68.3%, and 88.3% of the patients had sensory blockade, respectively. Comparison of the sensory blockade in the lateral part of the thigh at different time intervals indicates a significant p-value, as shown in Table 3.

**Table 3:** Sensory blockade in the lateral, medial, and anterior parts of the thigh at different time intervals P-value of <0.05 is considered significant

Time (min)	Lateral		Medial		Anterior	
	Yes	No	Yes	No	Yes	No
5	10 (16.7%)	50 (83.3%)	2 (3.3%)	58 (96.7%)	4 (6.7%)	56 (93.3%)
10	23 (38.3%)	37 (61.66%)	23 (38.3%)	37 (61.66%)	16 (26.7%)	44 (73.3%)
20	41 (68.3%)	19 (31.4%)	45 (75%)	15 (25%)	44 (73.3%)	16 (26.7%)
60	53 (88.3%)	7 (11.7%)	49 (81.7%)	11 (18.3%)	49 (81.7%)	11 (18.3%)
X^2^	72.69		94.58		94.77	
P-value	<0.001		<0.001		<0.001	

In the medial part of the thigh, 75% of the patients had a sensory blockade at the end of 20 minutes. The p-value comparing the sensory blockade in the medial part of the thigh at 20 minutes and at 60 minutes after the block was significant. Sensory blockade at the end of 60 minutes after the block was noted in 81.7% of the patients.

In the anterior aspect of the thigh, 6.7% of the patients had sensory blockade at the end of five minutes. At the end of 10 and 20 minutes, 26.7% and 73.3% of the patients had sensory blockage, respectively. A comparison of the sensory blockade in the anterior aspect of the thigh at 10 minutes and 20 minutes after the FICB indicates a significant p-value, as shown in Table 3.

Comfort of the anesthesiologists

The anesthesiologists were asked about the comfort while administering spinal anesthesia and positioning of the patients. It was observed that anesthesiologists had good comfort with 75% of the patients, and with 15% of the patients, the comfort was excellent.

Adverse events

It was observed that out of 60 patients, 10 patients experienced nausea as a side effect after the procedure. No serious complications like bladder injury or neurovascular damage were encountered in our study. Urine output and color of urine were observed to rule out bladder injury. Recovery of motor and sensory functions was assessed to rule out any nerve injury. Distal pulses were monitored to confirm that there was no vascular injury. 

## Discussion

In the context of femur fractures, patients necessitate comprehensive pain management from admission to final rehabilitation. Addressing optimal perioperative analgesia is crucial, particularly for surgeries like hemiarthroplasty and dynamic hip screw (DHS) placement for fractured neck of femur. The skin incision site is at the junction of the medial and lateral thigh, approximately 2 cm from the ASIS. This area's cutaneous distribution is covered by the femoral and lateral cutaneous nerve of the thigh. The femoral nerve supplies the retracted fascia lata and vastus muscles during the procedure, with a small part of the posterior acetabulum receiving innervation from the nerve to the quadratus femoris. With the introduction of ultrasonography, peripheral nerve blocks have become increasingly popular for lower limb surgeries [6]. The fascia iliaca compartment block (FICB) effectively blocks the femoral and lateral cutaneous nerve of the thigh, thus providing effective pre and postoperative analgesia in patients with fractured neck of femur, femoral shaft fracture, trochanteric fracture, and total hip replacement [7]. We conducted this observational study to investigate the analgesic efficacy of USG-guided FICB immediately after giving the block and during positioning for spinal anesthesia.

In a previous study by Yun et al. [8], 40 patients were divided into two groups receiving either FICB or intravenous analgesia. Visual analogue scores (VAS) were calculated during positioning for spinal anesthesia, and it was concluded that VAS scores were lower in patients who received FICB. In a similar study, Odor et al. studied the pharmacokinetic profile of levobupivacaine for fascia iliaca block in patients above 80 years [9]. They used 30 ml of 0.25% levobupivacaine. They did not observe any incidence of toxicity. The plasma levobupivacaine levels were below the threshold associated with toxicity. In our study, we also used 30 ml of 0.25% levobupivacaine for ultrasound-guided fascia iliaca compartment block one hour before spinal anesthesia and saw the efficacy of analgesia during positioning for spinal anesthesia using the NRS scale. Since the absorption of levobupivacaine is slow, at this concentration and volume, the plasma concentrations will be below the toxic threshold; hence, we did not observe any major complications during our study.

Several other studies have also used NRS as a pain assessment tool. Dochez et al. [10] studied the efficacy of pre-hospital administered fascia iliaca block in patients coming with hip fractures in the emergency department. They assessed the NRS score at the arrival, at five, 10, 20, and 30 minutes after the block. They found a 96% decrease in the pain score among the patients who received the FICB. Similarly, we calculated NRS before administering the block, 20 minutes after giving the block, in the patient holding area. After shifting to the operating room, NRS was again assessed during positioning for spinal anesthesia. We found that NRS was highest with 9.03% before the block. During positioning, it was 3.25%. The p-value obtained by comparing the NRS pre-block and during positioning was significant. Hence, we concluded that preoperative USG-guided FICB given one hour before the procedure effectively reduces the pain in patients while positioning for subarachnoid block (SAB).

In a similar study as ours, Kumar et al. [1] compared the sensory blockade in the lateral, medial, and anterior aspects of the thigh, and five minutes after giving the block, 18% of patients had sensory blockade compared to only 16.7% in our study. At 10-minute time interval, 80% of the patients had blockade compared to only 40% in our study. This early onset of action in their study could be because of the use of 30 ml of 0.5% ropivacaine compared to the use of 30 ml of 0.25% levobupivacaine in our study. While positioning for the spinal anesthesia, 11 patients out of 60 had NRS of more than seven at the time of spinal positioning, and rescue analgesia was given in the form of IV fentanyl 50 mcg. 

In a study performed by Levente et al. [11], a fascia iliaca compartment block was given in the emergency care unit. A volume of 40 mL of 0.5% ropivacaine was injected through USG guidance between the fascia iliaca and the iliopsoas muscle. If the patient weighed less than 70 kg, the local anesthetic volume was calculated for the maximum dose of 3 mg/kg. According to their study, the duration of analgesia after the fascia iliaca block was 48 hours. In our study, the USG-guided FICB was given one hour before the commencement of the surgery. A subarachnoid block was administered in the operating room. We used NRS to assess pain in our study at pre-block, 20 minutes after the block, and during positioning for subarachnoid block. Here, the scale ranged from 0-10. A score of more than seven indicated severe pain.

In a study conducted by Dolan et al. [12], a statistically significant increase in the sensory blockade was seen in patients with the use of ultrasound-guided technique as compared to landmark technique for femoral and obturator nerve blocks. Similarly, we used the ultrasound-guided fascia iliaca compartment block technique, which provided significant pain relief without any major complications. Anesthesiologists were comfortable 75% of the time while administering the subarachnoid block one hour after FICB.

Limitations

The success of the fascia iliaca compartment block may be influenced by the operator's skill and experience in performing the ultrasound-guided procedure. Variability in different practitioners' skills could affect the reproducibility of the results. In our study, we use 0.25% levobupivacaine for the fascia iliaca compartment block, limiting the exploration of potential variations in drug efficacy that might be observed with different local anesthetics or concentrations. Finally, the absence of blinding in assessing outcomes, such as NRS scores and anesthesiologist comfort, could introduce bias, as those involved might be aware of the intervention.

## Conclusions

The primary objective of this study was to evaluate the efficacy and safety of ultrasound-guided fascia iliaca compartment block in positioning patients for spinal anesthesia. The results of our study demonstrate that ultrasound-guided fascia iliaca compartment block provides adequate analgesia for positioning patients with hip fractures for spinal anesthesia in 81.6% when administered one hour prior to it. Pain relief provided by the block made it easy to position these patients for spinal anesthesia. Increased comfort of the anesthesiologists was noted while administering spinal anesthesia because of excellent pain relief in a significant number of patients without any noted major side effects of the ultrasound-guided fascia iliaca block or the drug 30 ml of 0.25% levobupivacaine used for the block. However, ultrasound-guided fascia iliaca compartment block is skill-dependent and may be difficult to reproduce in less experienced hands.
